# Dynamic nano-imaging of label-free living cells using electron beam excitation-assisted optical microscope

**DOI:** 10.1038/srep16068

**Published:** 2015-11-03

**Authors:** Masahiro Fukuta, Satoshi Kanamori, Taichi Furukawa, Yasunori Nawa, Wataru Inami, Sheng Lin, Yoshimasa Kawata, Susumu Terakawa

**Affiliations:** 1 Graduate School of Science and Technology, Shizuoka University, 3-5-1 Johoku, Naka, Hamamatsu 432-8561, Japan; 2 Institute of NanoScience Design, Osaka University, 1-3, Machikaneyamacho, Toyonaka 560-0043, Japan; 3 Research Institute of Electronics, Shizuoka University, 3-5-1 Johoku, Naka, Hamamatsu 432-8561, Japan; 4 CREST, Japan Science and Technology Agency, 4-1-8, Honmachi, Kawaguchi, Saitama 332-0012, Japan; 5 Photon Medical Research Center, Hamamatsu University School of Medicine, Hondayama, Higashi, Hamamatsu

## Abstract

Optical microscopes are effective tools for cellular function analysis because biological cells can be observed non-destructively and non-invasively in the living state in either water or atmosphere condition. Label-free optical imaging technique such as phase-contrast microscopy has been analysed many cellular functions, and it is essential technology for bioscience field. However, the diffraction limit of light makes it is difficult to image nano-structures in a label-free living cell, for example the endoplasmic reticulum, the Golgi body and the localization of proteins. Here we demonstrate the dynamic imaging of a label-free cell with high spatial resolution by using an electron beam excitation-assisted optical (EXA) microscope. We observed the dynamic movement of the nucleus and nano-scale granules in living cells with better than 100 nm spatial resolution and a signal-to-noise ratio (SNR) around 10. Our results contribute to the development of cellular function analysis and open up new bioscience applications.

Optical microscopes are effective tools for cellular function analysis because biological cells can be observed non-destructively and non-invasively in the living state in either water or atmosphere condition. Many optical microscopes such as phase-contrast, fluorescence, and confocal microscopes have been proposed, and many cellular functions analysed^1^,^2^,^3^,^4^. Because of the developments in nanotechnology, we can now observe the dynamics of fine structures in biological cells. For example, the sizes of proteins in the cells range from a few nanometres to a few tens of nanometres. In order to analyse the functions of these proteins, granules, and organelles, a higher resolution imaging technique is strongly required because the spatial resolution of a conventional optical microscope is limited to 200 nm by the diffraction limit of light.

Recently, the super-resolving fluorescent microscopes such as the stimulated emission depletion (STED) microscope^5^,^6^, structured illumination microscope (SIM)^7^,^8^, photo activated localization microscopy (PALM)^9^,^10^, and stochastic optical reconstruction microscopy (STORM)^11^,^12^ have been developed and widely used. And also electron microscope techniques have been applied to biological analysis^13^,^14^,^15^,^16^. These super-resolving microscopes have successfully analysed cells labelled with fluorescent dyes.

The decovolution technique is also effective to improve the spatial resolution of the observation image. The blur of observation image is removed by the application of the deconvolution technique. The decovolution technique is widely used in the fluorescence microscope such as confocal microscope and STED microscope^17^,^18^, and phase contrast microscopy^19^,^20^.

The 4Pi microscope also allows for high spatial resolution imaging of unlabeled specimen^21^ and the spatial resolution of the 4Pi microscope is limited by the diffraction limit of the light. The 4Pi has almost 100 nm in axial resolution, and 200 nm in lateral resolution^21^.

Here, we demonstrate the dynamic imaging of a label-free living cell at a high spatial resolution by using a nano-scale CL light source. We successfully observe the dynamics of the nucleus and cellular granules by scanning the nano-scale light source. The advantages of the label-free imaging over the labelling techniques are: 1) no requirements to prepare the biological sample before observation, 2) reduction of damage, deformation and artifacts of the biological sample caused by the labeling process, and 3) no need for photobleaching of the fluorescent dye^22^,^23^. Because of these advantages, the label-free imaging makes long-term observation of biological cells possible. It is effective for cellular function analysis and significant in the dynamic analysis of biological cells.

In previous work, we observed the label-free cells by using direct excitation of their auto fluorescence excited by the electron beam through the Si_3_N_4_ membrane^24^,^25^.

In the EXA microscope, the image contrast is given by the scattering or absorption of the CL by the microstructure in the biological specimen. Since the light absorption of cell differs depending on the cell type and cell structure^26^, the EXA microscope has potential to separate the microstructure in the cells by using refractive or absorption coefficient difference of each structure from spectroscopic imaging. The EXA microscope only observes at near the surface of thin film with high resolution.

Figure 1 shows the principle of the EXA microscope^27^. The nano-scale CL is excited on the luminescent film with a irradiating the focused electron beam. The spot size of the CL at the film surface is a few tens of nanometres in diameter because the electron beam can be focused to a few nanometres in diameter^28^. The CL light absorbed or scattered by the biological specimen on the film is detected with a high-sensitivity detector. The image of the biological specimen is obtained by raster scanning of the nano-scale CL light source. In the EXA microscope, the biological specimen can be observed under atmosphere condition because the luminescent film and the substrate separate the atmosphere condition from the vacuum condition. In this study, we use Zn_2_SiO_4_ for the luminescent film. The CL emission of the Zn_2_SiO_4_ is 300 nm, 380 nm, and 500 nm. We have succeeded to develop a Zn_2_SiO_4_ film of 20 nm thickness by annealing a ZnO film sputtered on a Si_3_N_4_ substrate at 1000 °C in nitrogen gas.

## Results

### High spatial resolution imaging of label-free cells with the EXA microscope

**Figure 2:**
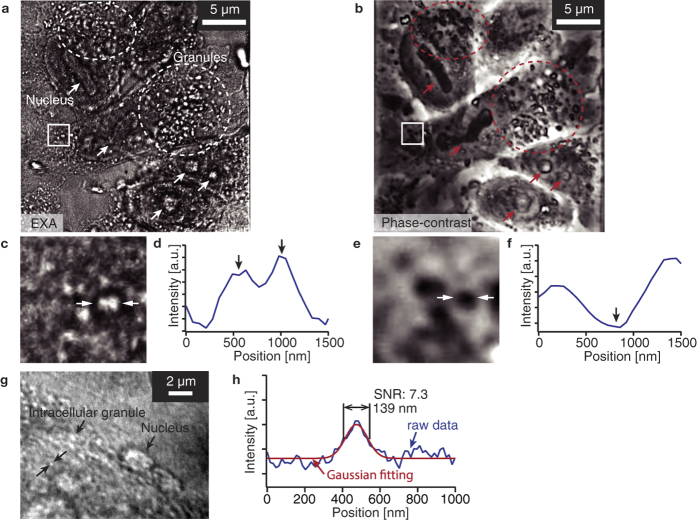
(**a**,**c**) EXA microscope image of the unstained MARCO cell. The nucleus and the intracellular granules are observed as bright spots. (**b**,**e**) Phase contrast microscope image of the MARCO cell. The phase contrast microscope image corresponds to the EXA microscope image as shown in Fig. 3(a,c,d,f). Intensity profile of intracellular granule indicated by arrows in Fig. 3(c,e,g). EXA microscope image of the unstained MARCO cell with higher magnification. (**h**) Intensity profile of a granule as shown in Fig. 3(g). The FWHM of the intensity profile is 139 nm.

Figure 2(a,b) shows the EXA microscope and conventional phase-contrast microscope image of the unstained macrophage receptor with collagenous structure (MARCO) cells. The MARCO cell was fixed by 1% glutaraldehyde and observed in a phosphate buffered saline (PBS) solution. The acceleration voltage of the electron beam and the irradiation current were 5 kV and 1 nA, respectively. The frame rate was 0.05 fps. The image size is 1024 × 1024 pixels. The cell nuclei are indicated with arrows and cellular granules are surrounded with circles. The nucleus and granules in the MARCO cell are observed as bright spots in Fig. 2(a). The unstained biological cells were successfully observed, with a higher resolution. Figure 2(c,e) are magnified images of the square region in Fig. 2(a,b). Figure 2(d,f) also show the intensity profile of cellular granules indicated by arrows in Fig. 2(c,e). It is clearly recognized that the EXA microscope can resolve the two granules in Fig. 2(c,d), while the phase-contrast microscope is unable to resolve them and observes them as a single particle (Fig. 2(e,f)). The EXA microscope observes the fine structures and the invisible structures in the phase-contrast microscope. The individual granules are clearly imaged in the EXA microscope observations.

We also evaluated the imaging resolution for the granules inside cells. Figure 2(g) shows the EXA microscope image of intercellular granules and the nucleus in an unstained MARCO cell. The MARCO cell was fixed by 1% glutaraldehyde and observed in a PBS solution. The acceleration voltage of the electron beam and the irradiation current were 5 kV and 1 nA, respectively. The frame rate was 0.05 fps. The image size is 1024 × 1024 pixels. Figure 2(h) shows the intensity profile of the intracellular granule between the two arrows in Fig. 2(g). The FWHM of the intensity profile is 139 nm. We achieve super-resolution imaging of the intracellular granule of the unstained MARCO cell by using the EXA microscope.

### Dynamic observation of label-free cells with the EXA microscope

**Figure 3:**
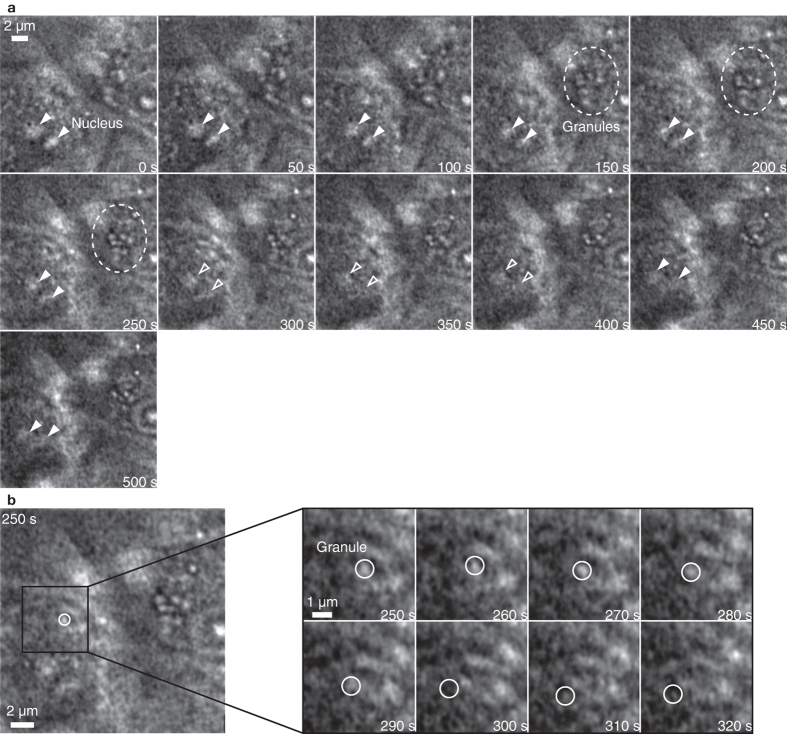
(**a**) Time-lapse image of the movement of the nuclei and the granules in the MARCO cell (Media 1). The nuclei move and disappear at 300 s, and reappear again at 450 s. The intracellular granules become concentrated in the circle region from 150 s. (**b**) Time-lapse image of the dynamic movement of the unstained single granule (Media 2). The granule moves in the left direction in the cell. The average velocity of the granule is 33 nm/s. The granule movement image size is 512 × 512 pixels, and the frame rate is 1 fps.

Figure 3(a) and Media 1 show the time lapse imaging of the nuclei and granules in the unstained living MARCO cell as captured by the EXA microscope. The image size was 512 × 512 pixels, the acceleration voltage was 5 kV, the irradiation current 1 nA, and the frame rate 1 fps. The nucleus and the granules in the unstained living MARCO cell are imaged as bright spots. It is possible to observe the dynamic movements of the nuclei and the granules in the time-lapse imaging. The nuclei, as indicated with the triangle-marks, disappeared at the time of 300 s, and they reappeared again at 450 s. This is due to the up-and-down movement of the nuclei, because only the region near the substrate can be imaged by the EXA microscope^23^. In Fig. 3(a), the nuclei stayed near the Si_3_N_4_ until 300 s. From 300 s to 450 s, the nuclei disappeared because they moved away from the Si_3_N_4_ substrate. From 450 s, it is also possible to observe the process that the nucleus comes close to the Si_3_N_4_ again and the nuclei are imaged. The intracellular granules are concentrated in the cell nucleus in the circle region at 150 s. At the time of 250 s, many granules are concentrated in the circle region.

Figure 3(b) and Media 2 show the magnified time-lapse image of an unstained granule. The granule is indicated by a circle. The granule moves in the left direction from 250 sec to 320 sec. The average velocity of a granule was 33 nm/s. The dynamics of the intracellular granules in the cell nucleus were observed in a label-free condition.

We calculated the average electron dose to discuss about the damage by electron beam irradiation. The average electron dose is commonly used for evaluation of electron beam irradiation damage to the cells.

In our experiment setup, the acceleration voltage is 5 kV, irradiation current is 1 nA, size of each pixel is 47 nm, and frame rate is 1 fps. From these experiment setups, the average electron dose for the dynamic observation of living cells was 11 electrons/nm^2^. This electron dose is much lower than other living cell observation reports by using electron microscope such as living yeast cell observation (22 electron/nm^2^)^29^, living COS-7 cell observation (300 electron/nm^2^)^30^, and living HeLa cells observation (42 electrons/nm^2^)^24^. According to these examples, we concluded that the cells are still living during observation and electron beam irradiation damage of the cells is very low in our experiment setup.

**Figure 4:**
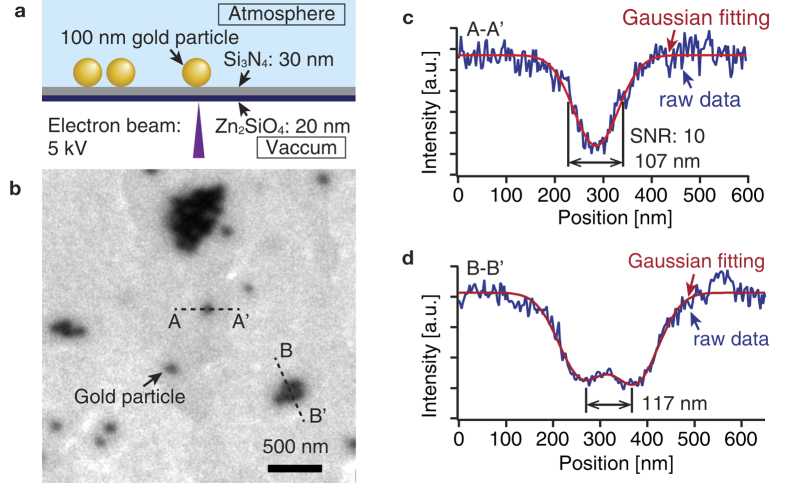
(**a**) Schematics of the spatial resolution estimation of the EXA microscope. The diameter of the gold nano-particles is 100 nm. The thicknesses of the Si_3_N_4_ and Zn_2_SiO_4_ layers are 30 and 20 nm, respectively. The acceleration voltage of the electron beam is 5 kV. (**b**) Observation result for the EXA microscope. The gold nano-particles are indicated as black points. (**c**) Intensity profile of the single gold nano-particle (A-A′) as shown in Fig. 3(b). The full width at half maximum (FWHM) of the intensity profile is 107 nm, and the SNR is 10. (**d**) Intensity profile of the neighbouring gold nano-particle (B-B′) as shown in Fig. 3(b). The gold nano-particles can be distinguished as single particles at a distance of about 117 nm. Thus, the EXA microscope using the Zn_2_SiO_4_ thin film has a sub-diffraction limit spatial resolution.

We evaluate the spatial resolution of the EXA microscope by observing gold nano-particles. The diameter of the gold nano-particles is 100 nm. Figure 4(a) shows the schematic of the film structure. The Zn_2_SiO_4_ film is formed on the backside of the Si_3_N_4_, opposite the specimen side. The gold nano-particles were dispersed on the Si_3_N_4_, and illuminated with CL from the Zn_2_SiO_4_. The thickness of the Si_3_N_4_ and the Zn_2_SiO_4_ layers were 30 and 20 nm, respectively. The acceleration voltage of the incident electron beam was 5 kV and the electric current was 1 nA.

Figure 4(b) shows the result of observing the gold nano-particles by the EXA microscope. The gold nano-particles are imaged as dark regions. Figure 4(c) shows the intensity profile along the line A-A′ shown in Fig. 4(b). The full width at half maximum (FWHM) of the intensity profile is 107 nm. With this result in Fig. 4(c), we may say that the EXA microscope has a spatial resolution beyond the diffraction limit of light (200 nm).

The SNR of the intensity profile in Fig. 4(c) is about 10. The SNR is calculated as the ratio of the height of the peak signal and the standard deviation of the background. According to the Rose criterion, the SNR is required to be more than 5 to reliably identify of an image object^31^,^32^. Hence, the dark regions in Fig. 4(b) provide reliable identification of the gold nano-particles.

Figure 4(d) shows the intensity profile along the line indicated with B-B′ in Fig. 4(b). In Fig. 4(d), the two side-by-side gold nano-particles were distinguished as two dips at a distance of 117 nm. Thus, we can confirmed that the spatial resolution of EXA microscopy is higher than the diffraction limit of light. The film structure used in this study also satisfies the requirements for achieving both of the high spatial resolution and high SNR.

## Discussion

We have observed the dynamic movement of an unstained biological cell with super resolution by using a nanometric scale CL light source generated by a focused electron beam. The unstained MARCO cells were cultured directly on the Si_3_N_4_ substrate. We also observed the movement of the intracellular granules and the nuclei of the unstained MARCO cell with 1 fps. In particular, the up-and-down movement of the nuclei and the concentration process of the granules in the cells were noticed. The spatial resolution of the EXA microscope using the Zn_2_SiO_4_ luminescent film was evaluated by observing 100 nm gold nano-particles. We confirmed that the spatial resolution of EXA microscope is better than 100 nm, while the SNR of the obtained image was 10. The EXA microscope is a very effective imaging technique for dynamics analysis of an unstained biological cell and helps to reveal the new cellular function.

## Methods

### Instrument setup

The EXA microscope is constructed by combination of fluorescent microscope (Olympus, BXFM) and scanning electron microscope (APCO, MINI-EOC)^23^. The Si_3_N_4_ substrate (Silson) and luminescent film separate the vacuum condition and atmosphere conditions. The CL excited in the luminescent film is detected by a photomultiplier tube (Hamamatsu Photonics K. K., H10721–110).

### Forming procedure of the Zn_2_SiO_4_ film

In this study, we use Zn_2_SiO_4_ for the luminescent thin film to improve the spatial resolution and SNR for dynamic imaging in the sub-diffraction limit. We have succeeded to develop a Zn_2_SiO_4_ film of 20 nm thickness by annealing a ZnO film of 50 nm thickness sputtered on a Si_3_N_4_ substrate at 1000 °C in nitrogen gas^33^,^34^. The luminescence of the light emitted by Zn_2_SiO_4_ is 20 times brighter luminescence than that of an as-deposited ZnO film even though the thickness is 20 nm, which is 5 times thinner than that of an as-deposited ZnO^35^. In addition, the Zn_2_SiO_4_ solves the charge-up problem caused by irradiating with the electron beam because it has good electrical conductivity.

### Sample preparation

The culture dish for the EXA microscope was composed of a glass dish, metal plate and Si_3_N_4_ substrate with the deposited luminescent film. The individual parts were fixed by epoxy resin. The size of the Si_3_N_4_ window is 50 *μ*m × 50 *μ*m. The biological cell was cultured directly on the Si_3_N_4_. The cells were incubated in a Dulbecco’s Modified Eagle’s Medium (DMEM, Sigma) until they adhered to the film. After incubation, the DMEM was removed and the phosphate buffer saline (PBS) was replaced in the culture dish before the observations.

## Additional Information

**How to cite this article**: Fukuta, M. *et al.* Dynamic nano-imaging of label-free living cells using electron beam excitation-assisted optical microscope. *Sci. Rep.* **5**, 16068; doi: 10.1038/srep16068 (2015).

## Supplementary Material

Supplementary Information

Supplementary Media 1

Supplementary Media 2

## Figures and Tables

**Figure 1:**
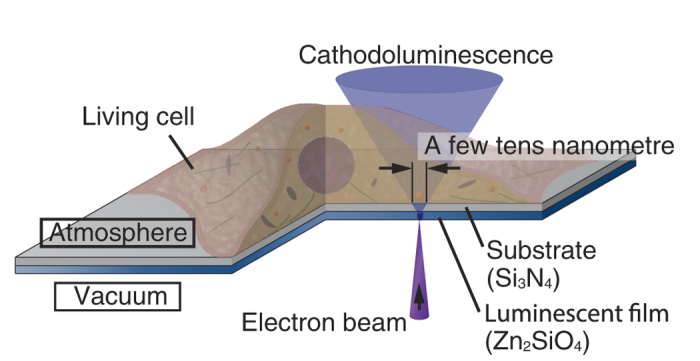
Principle of the electron beam excitation-assisted optical (EXA) microscope. The focused electron beam irradiates the luminescent film, exciting the nano-scale cathodoluminescence (CL). The specimen is observed by scanning the tiny CL light source. The living cell is placed on to the Si_3_N_4_ substrate in atmosphere condition.
