# Supplementary material for: Dynamic nano-imaging of label-free living cells using electron beam excitation-assisted optical microscope
Source: Sci Rep. 2015 Nov 3;5:16068. doi: 10.1038/srep16068 (PMC4630636; doi:10.1038/srep16068)
Supplement: Supplementary Information [file srep16068-s1.doc]

**Title**

**Dynamic nano-imaging of label-free living cells using electron beam excitation-assisted optical microscope**

**Masahiro Fukuta^1^, Satoshi Kanamori^1^, Taichi Furukawa^2^, Yasunori Nawa^3^, Wataru Inami^3, 4^, Sheng Lin^4^, Yoshimasa Kawata^3, 4, *^, and Susumu Terakawa^4, 5^**

^1^*Graduate School of Science and Technology, Shizuoka University, 3-5-1 Johoku, Naka, Hamamatsu 432-8561, Japan*

^2^*Institute of NanoScience Design, Osaka University, 1-3, Machikaneyamacho, Toyonaka 560-0043, Japan*

^3^*Research Institute of Electronics, Shizuoka University, 3-5-1 Johoku, Naka, Hamamatsu 432-8561, Japan*

^4^*CREST, Japan Science and Technology Agency, 4-1-8, Honmachi, Kawaguchi, Saitama 332-0012, Japan*

*^5^Photon Medical Research Center, Hamamatsu University School of Medicine, Hondayama, Higashi, Hamamatsu*

**Supplementary Information**

**Movie legends**

Media 1. Movement of the nuclei and the granules in the MARCO cell. The nuclei move and disappear at 300 s, and reappear again at 450 s. The intracellular granules become concentrated in the circle region from 150 s.

Media 2. Dynamic movement of the unstained single granule. The granule moves in the left direction in the cell. The average velocity of the granule is 33 nm/s.

**Schematics of the culture dish for the EXA microscope**

Figure 1 shows the schematic diagram of the culture dish for the EXA microscope. The glass dish, metal plate and substrate make up the culture dish of the EXA microscope. The living cell is cultured directly on the Si_3_N_4_. The Zn_2_SiO_4_ is deposited under the Si_3_N_4_.

Figure 1. Schematics of the culture dish for the EXA microscope.
